# Assessing violations of the proportional hazards assumption in Cox regression: does the chosen method matter?

**DOI:** 10.1186/1745-6215-16-S2-P134

**Published:** 2015-11-16

**Authors:** Louise Hiller, Andrea Marshall, Janet Dunn

**Affiliations:** 1 University of Warwick, Coventry, UK

## Objectives

The Cox proportional hazards (PH) model is commonly used in randomised clinical trials (RCTs) to assess a treatment effect after adjusting for known prognostic factors. However, the Cox model requires that a covariate effect is constant over time. Violation of this assumption invalidates the simple Cox model. Various PH checks exist, some in the form of statistical tests and some empirical ones involving graphical examination. We investigated if results vary from some of the different methods available.

## Methods

Individual patient data for the same five prognostic factors was collated from 6248 patients participating in four similar RCTs and a time to event analysis undertaken (median follow-up 6.2 years; 1335 events). The PH assumption was checked for each variable using four different methods:

1/ Plot of the log of the negative log of the estimated survival density function vs log(time)

2/ Cumulative Sums of Martingale Residuals and the Kolmogorov-type Supremum Test

3/ Fitting time dependent covariates

4/ Schoenfeld's residuals

## Results

For two variables, all four PH assessment methods agreed. For the other three variables, different results were found depending on the method used.

## Conclusion

Different conclusions were reached on PH violation of covariates depending on which PH assessment method was used. With the requirement for upfront Statistical Analysis Plans (SAPs) specifying the exact statistical methodology for analysing RCTs, it is also recommended that the PH assessment technique to be used to determine the validity of the Cox PH model should be stated within RCT SAPs.

